# Influence of lip volume enhancement with hyaluronic acid in-filtrations on lip Imprints

**DOI:** 10.4317/jced.62395

**Published:** 2025-01-01

**Authors:** Laura Luis-Sanchez, Daniel Torres-Lagares, Celia Vazquez-Pachón, Esther Hernandez-Pachecho, Gonzalo Ruiz-de-Leon-Hernandez, Maria Angeles Serrera-Figallo, Jose Luis Gutierrez-Perez, María Baus-Dominguez

**Affiliations:** 1 Faculty of Dentistry, University of Seville, C/Avicena s/n, 41009 Seville, Spain

## Abstract

**Background:**

Cheiloscopy, as a method of forensic identification, maintains its currency in legal and forensic dentistry. The increase in aesthetic lip filler treatments could influence lip prints. This aspect has not yet been studied to the research team’s knowledge. Objective: This study aims to evaluate the possible modification of lip imprints after hyaluronic acid infiltration for volume augmentation in this location.

**Material and Methods:**

A descriptive, non-experimental study was carried out in patients with lip augmentation using hyaluronic acid infiltration (32 patients). Pre-infiltration data were collected (age, sex, lip traces, and lip characteristics), and data on the infiltration performed (volume used, points, and infiltration technique). During five visits, the evolution of the lip print and lip characteristics was monitored six months after the initial infiltration.

**Results:**

Applying the Suzuki and Tsushihashi classification, 92.9% of the cases studied showed differences concerning the preoperative situation.

**Conclusions:**

From the data of the present study, we can affirm that, without generating critical changes, lip hyaluronic acid infiltration modifies some characteristics of the lip print, which should be known and considered when using cheyloscopy as a forensic identification tool.

** Key words:**Lip prints, Hyaluronic acid, Cheiloscopy, Forensic identification, Semi-permanent in-filtration, Volume enhancement.

## Introduction

The labial mucosa presents a series of deep vertical grooves or folds whose morphology and distribution determine the formation of variable patterns (1).

These folds occupy the entire length of the lower mucosal lip, while in the upper one, they are arranged on both sides of the labial tubercle. The importance of these drawings is such that most authors consider that the study of the lips focuses exclusively on the analysis of these drawings and does not include the individual variations of other lip elements, such as thickness or shape (2).

Lip prints are the impressions left by the lips in contact with a surface and can be visible when the lips are stained (generally from cosmetic products) or latent when coated with saliva; they are of great value because they contain genetic material (3). It can also be interpreted in a strict sense, in which case, the term “cheyloscopy” would only deal with the study of the mucosal lip grooves and the traces left by them (the latter being the criterion used in the present investigation) (4,5).

The identification of lip prints has been equated with dactyloscopic identification because their characteristics as an identifying record are similar. Thus, their study is valid for identifying people. The characteristics of the lip prints are unique (it is accepted that no two prints are alike except for monozygotic twins), permanent (from the formation of the lips between the fourth and fifth month of intrauterine life, the lip furrows remain invariable in shape and location throughout the life of the individual) and invariable (it has been proven that the characteristics of the lips in their mucosal portion are wholly recovered after suffering alterations and pathologies of the lips such as scars, herpes, etc., and that the arrangement and shape of the lip furrows remain invariable throughout the life of the individual), and that the arrangement and shape of the grooves do not vary due to environmental factors). For all these reasons, they are considered immutable (6-8).

There has been a significant increase in the demand for aesthetic lip treatments focused on modifying its shape and volume through infiltration with hyaluronic acid. This biological polymer, once introduced into the thickness of the lip tissue, begins to capture water and take on volume, thickening the lips and modifying their profile. Since the maintenance of hyaluronic acid at the infiltration site is temporary (about six months), the changes produced are not permanent (9,10).

This study aims to evaluate the possible modification of lip imprints after hyaluronic acid infiltration to increase the volume in this location. This study affects a dental area of great relevance, such as forensic dentistry. Variations in some of the characteristics of the lip prints after the aesthetic infiltration of the lips can cause difficulties in interpretation, increasing the susceptibility to possible errors in the identification of these prints (2).

## Material and Methods

-Type of Study 

This research is a prospective descriptive study. The Ethics Committee approved this study with the code CEIm HM Hospitales 23.11.2258-GHM, and it complies with all the guidelines of the World Medical Association Declaration of Helsinki: Ethical Principles for Medical Research Involving Human Subjects (11).

-Patient selection 

The study subjects were patients of both sexes and legal ages who met every one of the inclusion criteria and none of the exclusion criteria. The inclusion criteria included that the patients should be treated at the Faculty of Dentistry of Seville and that the patient should agree to participate in the study (informed consent); if they did not agree, the patient would be treated in the same way, but without taking the necessary data for the study, they should not present any scarring on the lip mucosa, they should not have received previous lip filling treatment, and they should not be immunosuppressed patients or have any autoimmune disease.

On the other hand, the exclusion criteria were patients allergic to hyaluronic acid gel, pregnant or breastfeeding women, patients in whom the status of any systemic disease itself or its treatment could affect our study (diabetes, hypertension, hematological disorders), and finally, failure to meet one or all the inclusion criteria.

After the indication of this treatment and acceptance by the patients, it was explained to them the possibility of entering this descriptive follow-up study, its objectives, and, above all, that taking records is innocuous and does not add risks to the treatment itself. After signing the informed consent form, the infiltration was carried out following the intervention protocol, which consisted of lip infiltration with hyaluronic acid using the technique described below (this protocol only differs from the protocol of usual clinical use in the taking of records). Finally, the lip impression was taken (12).

-Infiltration technique 

The infiltration protocol began with disinfection of the area with 0.12% chlorhexidine mouthwash (Lacer®, Barcelona, Spain), anesthesia of the region with infiltrative anesthesia in the vestibule of the lips with lidocaine 2% with epinephrine 0.05% (Normon®, Madrid, Spain). The areas necessary for adequate aesthetic results were infiltrated with hyaluronic acid in the labial submucosa. Finally, we massaged so that no palpable nodules remained.

In this study, both the linear technique, which consists of introducing the needle parallel to the dermis at the appropriate length and injecting small amounts of hyaluronic acid along the lip line in a linear fashion, and the depot technique, which consists of injecting tiny threads of hyaluronic acid into the most superficial layers of the lips with an excel-lent needle, are used. Combining both methods is called the combined technique (13,14).

The hyaluronic acid used in this study was Apriline® Normal (Osteogenos, Madrid, Spain), of non-animal origin. Each ml of isotonic solution contains 23 mg/ml of sodium hyaluronate, sodium chloride, sodium mono-hydrogen phosphate, sodium dihydrogen phosphate, and water for injections. The solution is presented in a graduated, pre-filled, single-use syringe. This study used a maximum of 1 ml of hyaluronic acid gel.

-Recording for cheyloscopy

Patient follow-up and record-taking were carried out during five visits, including pre-operative, one week, one month, three months, and finally, six months after the treatment started, to observe the changes in the patients’ lip prints.

This study emphasizes the classification of Suzuki and Tsushihashi (12) as it is one of the main methods used in cheiloscopy and studies fundamentally the lip impressions for human identification based on the shape and course of the lip grooves and is divided into five primary types: type I or vertical grooves that cross the lip vertically, type II where the grooves bifurcate in their course, type III where the grooves cross, the reticulated form or type IV and finally the indeterminate form or type V, which is when the grooves do not belong to any of the types I-IV and cannot be differentiated morphologically.

**Figure 1:**
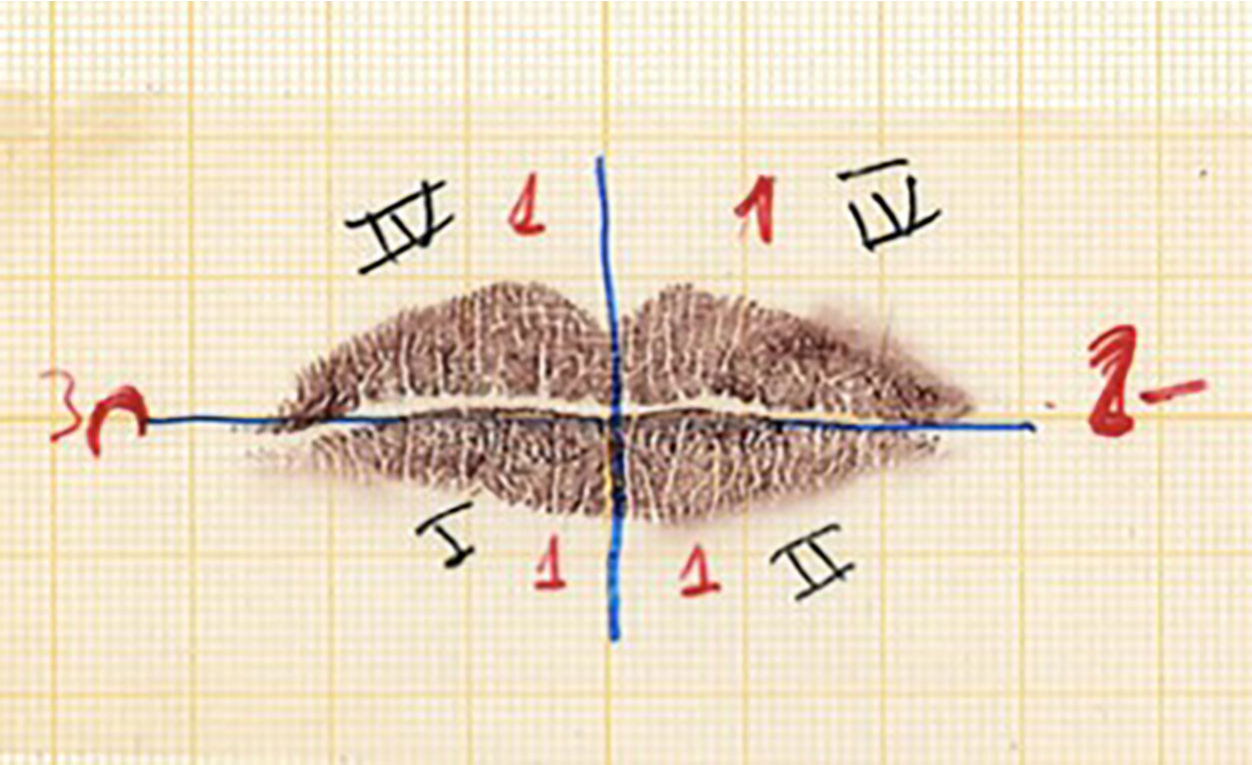
Recording for cheyloscopy.

The protocol for taking lip prints was as follows: lipstick (Superstay 24h Lipstick, Maybelline®, NY, USA) was applied on the mucosal part of the lips and left to act for five minutes. Then, a lip impression was printed on the paper to remove the excess, and finally, four impressions were made by contacting the lips on the chosen surface (graph paper, Liderpapel®, Malaga, Spain). After the impressions, photographs were taken to document the variables studied more faithfully (Fig. 1).

When taking the impressions, the impression was considered excellent when the contour of the lip print and the grooves of the mucosal lip were perfectly observed, both in the upper and lower lip. The impression was good if the lip contour was distinguishable and the commissure was identified. Not all the mucosal lip patterns were visible, but some grooves were observed. The print quality was poor when only the lip outline could be identified. All the prints included in this study were of excellent quality (15-18).

-Variables studied

The following variables were evaluated. Preoperatively, lip imprints were taken (using Suzuki and Tsushihashi’s classification (12), dividing the mouth into four sectors and assessing in each of them the existence of vertical, branched (Y), cross-linked, reticulated, or indeterminate grooves), upper and lower, right and left lip thickness (in mm), and labial commissures (vertical, horizontal, or downturned).

Intraoperatively, data were collected on the amount of hyaluronic acid infiltrated (by quadrants and total, in mm), the infiltration points (in upper lip, philtrum column, cupid’s bow (right or left), labial tubercle (right or left), labial profile (right or left), labial thickness (right or left), or commissure (right or left), in lower lip (labial thickness (right or left), labial profile, vermilion (right or left)), the technique used by labial quadrants (linear, deposit or combined).

Postoperatively, lip traces, commissures, and lip thickness were assessed at two weeks, one month, three months, and six months post-infiltration.

-Statistical analysis 

SPSS software (version 29 for Windows; IBM®, NY, USA) was used for statistical analysis. The data were presented in frequency distribution Tables. The chi-square test was used to evaluate whether the differences between qualitative variables were significant, and the Mann-Whitney U test was used for quantitative measures. All tests were performed at a statistical significance level of 0.05.

## Results

-Sample size

Only 32 patients were studied, although two did not complete the entire follow-up, so the final sample consisted of 30 patients (0 men, 30 women, mean age 32.59 years ± 13.3). The data relating to the techniques applied can be seen in  Table 1 and  Table 2.

-Infiltrated areas

Regarding the upper lip, the areas that were infiltrated by the most significant number of patients were the labial thickness, with 76.7% for the right side and 83.3% for the left side, followed by infiltration in the labial tubercle, both right and left with 66.7% and 73.3% respectively.

When we analyze the lower lip, we see that 53.3% of the subjects infiltrated the right and left vermilion. Only between 30 and 33% infiltrated the labial thickness, unlike the upper lips, which was the most significant number of patients.

Finally, neither the right nor left commissures received infiltration, and 100% of subjects did not receive infiltration in these areas.

-Technique used

The most used technique for the right and left upper lip was the combined technique, with 64.3% and 66.7%, respectively. However, this was not the case for the lower lip, where the linear technique predominated with 57.9% for both sides.

-Amount of hyaluronic acid infiltrated

When analyzing the data on the amount of hyaluronic acid placed, we observed that an average volume of about 0.31 ml infiltrated each upper lip and 0.27 ml in each lower lip. The total volume infiltrated, which includes all lips, was about 0.94 ml on average. However, it is essential to note that the volume infiltrated in the lower lips has a higher standard deviation, suggesting more significant variability in the volume infiltrated in this area.

Evolution of the labial commissures and labial thickness 

 Table 3 describes the evolution of the patterns in the lip prints throughout the follow-up period, while  Table 4 identifies the evolution of the commissures and lip thickness.

**Figure 2:**
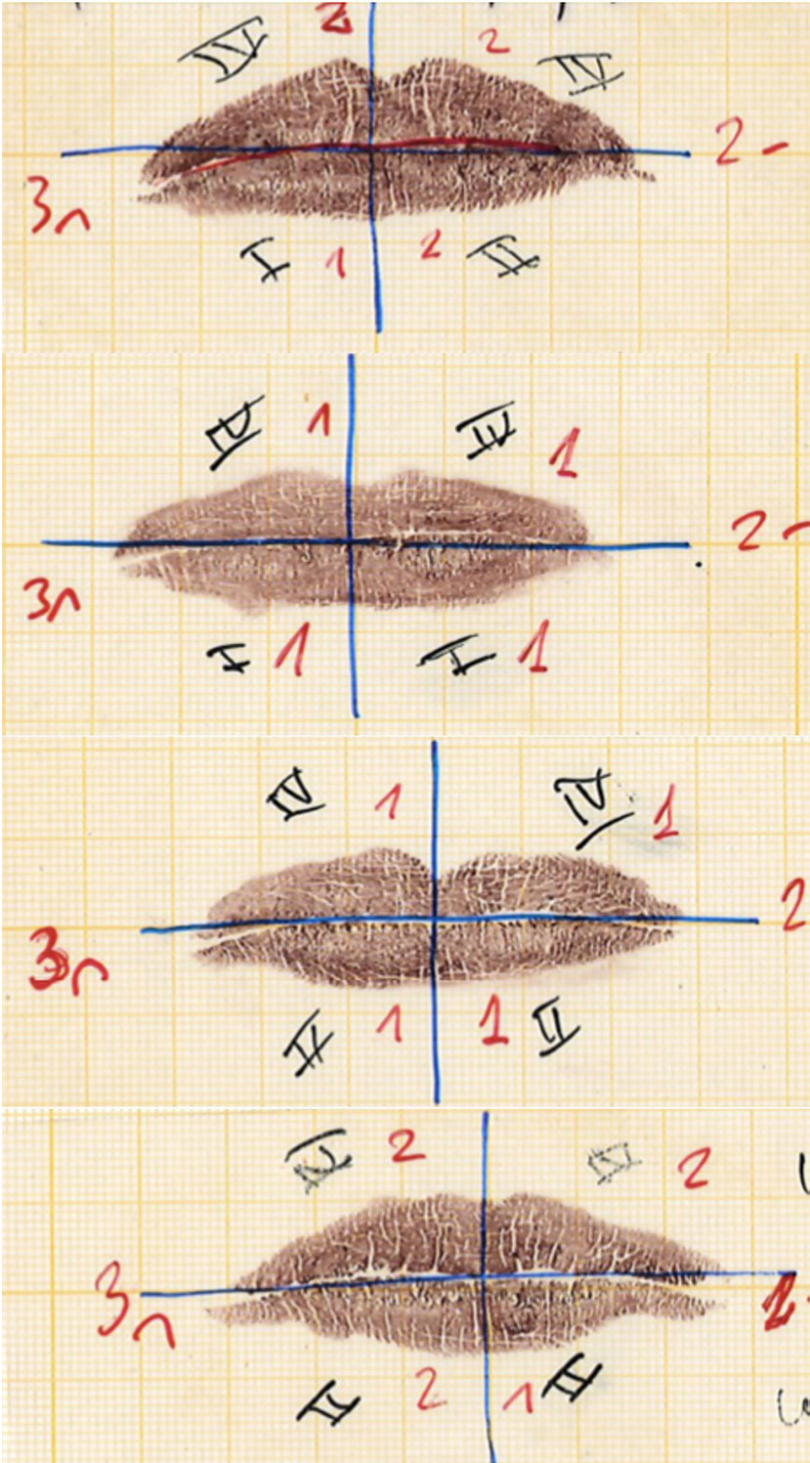
Evolution of labial commissures and labial thickness by analysis of rhinoscopy using the Suzuki and Tsushihashi Classification.

The data from the first visit showed that in the first and second quadrants of the right upper lip, the most frequent types of grooves were Type II (ramified) with 43.3% and 46.7%, respectively, followed by Type I (vertical grooves), with 23% and 26.7% respectively, while in the fifth visit, on the contrary, ramified grooves (Type II) decreased to 17.9% in the two quadrants. In the third and fourth quadrants, during the first data collection, in the lower lip, Type II was, likewise, the most prevalent, with 50% for the left quadrant and 46.7% for the right quadrant, followed by Type I with 33.3% and 36.7% respectively, on the contrary, at the end of the monitoring in the fifth visit Type II fur-rows continued to be the most frequent with 57.1% for both quadrants in the lower sector. The rest of the types of grooves behaved relatively the same during this process (Fig. 2).

Regarding the thickness of the lips and the commissures, we noticed that in the upper sector, between 60% and 70% of the subjects had thin lips; however, in the lower lips, patients with medium-thickness lips predominated, with 36.7% for the right side and 26.7% for the left lip. In other words, during data collection in this study, patients with thin upper lips and medium-thick lower lips predominated. After the treatment, already in the fifth visit of the patients, we noticed that most of the upper lips were no longer thin but of medium thickness (between 57.1% and 60.7% and in the lower sector, they continued to be medium thickness, but in a higher percentage (60%).

About commissures, there is a general predominance of downturned commissures, 76.7% on the right side, and horizontal commissures, 26.7% on the left side. In the last visit, we saw that the right side continued to be dominated by downturned commissures, but to a greater extent, with 71.4%, and the same is true for the left side, where horizontal commissures predominate, with 46.4%.

-Observed changes in global reference lip patterns, lip thickness, and commissures from baseline and at follow-up

 Table 5 presents the differences in lip imprints, commissures, and lip thickness throughout the follow-up time, concerning the preoperative situation.

When we analyzed the evolution of the changes in the time elapsed between the first visit and the fifth and last visit of the patients, we observed that for the Suzuki and Tsushihashi classification in the first quadrant of the right upper lip, there was a change in 40% of the cases during the first visit, however, already in the fifth visit the change corresponds to 64.3%. In the second quadrant of the left upper lip, 36% of the cases experienced changes at the first visit, which increased until the last record with 53.6%. When we study the third quadrant of the lower left lip, we see that the first data collection corresponds to 24% of changes, a Figure that reaches its most excellent modification between the first and third visit with 54.2%, because in general between the first and last visit, this quadrant undergoes minor changes, with 35.7%. In the fourth quadrant of the right lower lip, there were changes in 40% of the cases between the first and second visit; however, between the first and fourth visit, there was a higher percentage of changes, and finally, in the last data collection, there were modifications of 39.3%.

Regarding the thickness of the lip, in both the right and left upper lip, 44% of cases experienced changes, a Figure that remained relatively high until the last visit, with 39.3% and 57.1%, respectively. For the thickness of the left and right lower lip, there were changes in 28% and 32% of the cases, which in the rest of the visits reached between 41% and 50%, but finally, between the first and fifth revisions, changes of 17.9% for the left lower lip and 42.9% for the right lower lip were observed.

Between the first and last visits, the right commissure changed by 42.9%, and the left commissure changed by 35.7%.

-Changes between visits grouped by type of variable

 Table 6summarizes the changes between all the visits grouped by the type of variables studied. According to the Suzuki and Tsushihashi classification, we have a progressive increase in the data according to the visits; between the first and second visit, 64.0% of the cases showed changes, and between the first and third visit, there was a modification of 79.2% of the cases, between the first and fourth visit, the transformations were 85.7% of the cases, and finally between the first and fifth visit, 92.9% of the cases showed changes.

When analyzing the lip thickness, 80.0% of the cases showed changes between the first and second visits, 79.2% of the cases were modified between the first and third visits, 82.1% of the cases showed transformations between the first and fourth visits, and 78.6% of the cases showed changes between the first and fifth visits.

According to the commissions, there were upward changes of 48.0%, 45.8%, 46.4%, and 60.7% during the first, second, third, fourth, and fifth visits.

## Discussion

As mentioned above, cheyloscopy, within forensic science, studies lip furrows and lip prints (individuals except in uni vitelline twins) for identification purposes (18,19). Lips present lines, fissures, and combinations of these two forms specific to each person (20). Lip infiltration with hyaluronic acid can modify these lip imprints.

The possible modification of lip prints is one of the most evaluated parameters since it can decrease the reliability of this identification method in criminology. Based on our study, the forensic community would find new data on the increase in lip thickness, variation of lip prints, or commissures because of hyaluronic acid infiltration, which is increasingly frequent in our field due to its high demand (21,22).

When analyzing the patients in this study according to the classification of Suzuki and Tsushihashi (12), during the first data collection, we observed that there is a predominance of branched lip patterns in all quadrants, followed by vertical furrows, cross-linked, cross-linked, and indeterminate patterns had a lower presence compared to the previous ones. However, after hyaluronic acid infiltration at the fifth visit, vertical furrows (Type I) predominate for the upper lips and branched lip patterns (Type II) for the lower lips. This information shows that hyaluronic acid infiltration should be evaluated when cheyloscopy is a determining tool in forensic investigations (23).

Lip thickness is a characteristic that has been performed mainly as an indicator of skin color phenotype or racial indicator (24); when studying our patients (all of the Caucasian race) at the first visit, we observed significant variability in lip thickness among subjects, most had thin lips, followed by medium thickness, not being so at the end of treatment, since almost all patients had medium thickness lips and thus significantly increased the number of patients with thick lips. In addition, there is a tendency toward downturned commissures on both sides, with minimal vertical commissures. These findings may be helpful in forensic analysis to identify and differentiate individual lip characteristics of the subjects.

The site of hyaluronic acid placement may cause variability in cheyloscopy analysis. In this study, most upper lip infiltrations were performed in the tubercle and labial thickness, whereas the commissures did not receive infiltration in this sample. However, most of the acid was deposited in the vermilion (not in the profile or labial thickness) on both the right and left sides (25).

Interpretation of these findings may help understand patient preferences for locations of hyaluronic acid infiltration into the lip during cosmetic or reconstructive treatment and, likewise, understand that hyaluronic acid causes changes in lip thickness and, with this, in lip imprints, which are essential patterns to consider in a forensic investigation (13,25,26).

Assessing the technique applied when placing the acid on the lips (14,25,27). In our sample, the combined technique was the most frequently used, followed by the linear and deposition techniques.

Similarly, the amount of acid that is placed also influences many aspects; the greater the amount placed, the more significant the change in the lips; therefore, the more everyone’s features and lip folds will be lost. In this study, the amount of infiltration points and the infiltrated volume are within the average ranges expected for this type of procedure. However, the variability in the infiltrated volume in the lower lips is more significant, which may indicate a greater diversity in the needs or preferences of patients in that area.

This study evaluates whether one of the most common orofacial harmonization techniques, lip filler with hyaluronic acid, can modify the cheyloscopy or lip print to understand this forensic evidence better. Our data indicate that when hyaluronic acid is infiltrated to give more volume to the lips, the lip print is altered in some of its variables: drawing, morphology, dimension, or depth (28-30).

Table 6 shows that up to 92% of patients presented significant changes in lip patterns during this type of treatment. The mean number of changes tended to increase as time after infiltration increased, which could be caused by the labial surface accommodating the hyaluronic acid infiltration.

## Conclusions

From the data of the present study, we can affirm that, without generating critical changes, lip hyaluronic acid infiltration modifies some characteristics of the lip print (in up to 92% of the cases studied), which should be known and considered when using cheyloscopy as a forensic identification tool.

## Figures and Tables

**Table 1:** First visit. Location of hyaluronic acid infiltration in the upper and lower lip.

Zones	Location	Categories	Frequency	Percentage
Lower lip	Filtrum column	Yes	4	13.3
		No	26	86.7
	Cupid's bow (Right)	Yes	8	26.7
		No	22	73.3
	Cupid's bow (left)	Yes	10	33.3
		No	20	66.7
	Labial tubercle (Right)	Yes	20	66.7
		No	10	33.3
	Labial tubercle (left)	Yes	22	73.3
		No	8	26.7
	Lip profile (right)	Yes	14	46.7
		No	16	53.3
	Lip profile (left)	Yes	15	50.0
		No	15	50.0
	Lip thickness (right)	Yes	23	76.7
		No	7	23.3
	Lip thickness (left)	Yes	25	83.3
		No	5	16.7
	Commissures (Right)	Yes	0	0.0
		No	30	100.0
	Commissures (Left)	Yes	0	0.0
		No	30	100.0
Upper lip	Lip thickness (right)	Yes	9	30.0
		No	21	70.0
	Lip thickness (left)	Yes	10	33.3
		No	20	66.7
	Lip Profile	Yes	4	13.3
		No	26	86.7
	Vermilion (Right)	Yes	16	53.3
		No	14	46.7
	Vermilion (Left)	Yes	16	53.3
		No	14	46.7

**Table 2:** Preoperative visit. The technique used and volume infiltrated.

Zones	Variables (technique used)	Frequency	Percentage
Right upper lip	Linear	9	32.1
	Deposits	1	3.6
	Combined	18	64.3
Left upper lip	Linear	8	26.7
	Deposits	2	6.7
	Combined	20	66.7
Right lower lip	Linear	11	57.9
	Deposits	6	31.6
	Combined	2	10.5
Left lower lip	Linear	11	57.9
	Deposits	6	31.6
	Combined	2	10.5
Variables (Infiltrate Volume, ml)	No. of cases	Mean	S.D.
Infiltrate volume (right upper lip)	28	0.31	0.26
Infiltrated volume (upper left lip)	30	0.31	0.26
Infiltrate volume (right lower lip)	19	0.27	0.44
Infiltrate volume (Left lower lip)	19	0.27	0.44
Total infiltrated volume	30	0.94	1.15

**Table 3:** Global reference lip patterns. Suzuki and Tsushihashi classification in the preoperative visit and the different follow-up visits (F) Frequency (%) Percentage).

		Preoperative		15 days		One month		Three months		Six months	
Zone	Categories	(F)	(%)	(F)	(%)	(F)	(%)	(F)	(%)	(F)	(%)
First quadrant (upper lip - right)	Type I - Vertical grooves	7	23.3	12	48.0	12	50.0	12	42.9	15	53.6
	Type II - Branched (Y)	13	43.3	7	28.0	6	25.0	9	32.1	5	17.9
	Type III - Cross-linked	3	10.0	1	4.0	1	4.2	1	3.6	2	7.1
	Type IV - Reticulated	4	13.3	3	12.0	1	4.2	3	10.7	3	10.7
	Type V - Indeterminate	3	10.0	2	8.0	4	16.7	3	10.7	3	10.7
Second quadrant (upper lip - left)	Type I - Vertical grooves	8	26.7	13	52.0	15	62.5	12	42.9	15	53.6
	Type II - Branched (Y)	14	46.7	6	24.0	4	16.7	9	32.1	5	17.9
	Type III - Cross-linked	2	6.7	2	8.0	2	8.3	3	10.7	1	3.6
	Type IV - Reticulated	4	13.3	2	8.0	1	4.2	2	7.1	3	10.7
	Type V - Indeterminate	2	6.7	2	8.0	2	8.3	2	7.1	4	14.3
Third quadrant (lower lip - left)	Type I - Vertical grooves	10	33.3	6	24.0	12	50.0	10	35.7	9	32.1
	Type II - Branched (Y)	15	50.0	15	60.0	10	41.7	14	50.0	16	57.1
	Type III - Cross-linked	1	3.3	1	4.0	0	0.0	2	7.1	1	3.6
	Type IV - Reticulated	2	6.7	2	8.0	1	4.2	0	0.0	0	0.0
	Type V - Indeterminate	2	6.7	1	4.0	1	4.2	2	7.1	2	7.1
Fourth quadrant (lower lip - right)	Type I - Vertical grooves	11	36.7	8	32.0	10	41.7	8	28.6	8	28.6
	Type II - Branched (Y)	14	46.7	11	44.0	11	45.8	16	57.1	16	57.1
	Type III - Cross-linked	1	3.3	2	8.0	0	0.0	1	3.6	1	3.6
	Type IV - Reticulated	2	6.7	3	12.0	0	0.0	1	3.6	0	0.0
	Type V - Indeterminate	2	6.7	1	4.0	3	12.5	2	7.1	3	10.7

**Table 4:** Analysis of the thickness of the lips and labial commissures in the pre-operative visit and the different follow-up visits (F) Frequency (%) Percentage).

		Preoperative		15 days		One month		Three months		Six months	
Zone	Categories	(F)	(%)	(F)	(%)	(F)	(%)	(F)	(%)	(F)	(%)
Right upper lip thickness	Thin lips < 8 mm	18	60.0	7	28.0	5	20.8	8	28.6	8	28.6
	Lips average thickness 8 mm to 10 mm	11	36.7	17	68.0	13	54.2	14	50.0	16	57.1
	Thick lips >10 mm	1	3.3	1	4.0	6	25.0	6	21.4	4	14.3
Upper left labial thickness	Thin lips < 8 mm	21	70.0	8	32.0	5	20.8	10	35.7	7	25.0
	Lips average thickness 8 mm to 10 mm	8	26.7	16	64.0	15	62.5	10	35.7	17	60.7
	Thick lips >10 mm	1	3.3	1	4.0	4	16.7	8	28.6	4	14.3
Lower left labial thickness	Thin lips < 8 mm	10	33.3	9	36.0	7	29.2	9	32.1	7	25.0
	Lips average thickness 8 mm to 10 mm	15	50.0	12	48.0	14	58.3	14	50.0	17	60.7
	Thick lips >10 mm	5	16.7	4	16.0	3	12.5	5	17.9	4	14.3
Right lower lip thickness	Thin lips < 8 mm	11	36.7	11	44.0	9	37.5	10	35.7	7	25.0
	Lips average thickness 8 mm to 10 mm	13	43.3	10	40.0	12	50.0	13	46.4	17	60.7
	Thick lips >10 mm	6	20.0	4	16.0	3	12.5	5	17.9	4	14.3
Right commissure	Vertical (elevated)	0	0.0	0	0.0	1	4.2	0	0.0	1	3.6
	Horizontals	7	23.3	6	24.0	6	25.0	9	32.1	7	25.0
	Abatidas	23	76.7	19	76.0	17	70.8	19	67.9	20	71.4
Left commissure	Vertical (elevated)	2	6.7	0	0.0	0	0.0	0	0.0	4	14.3
	Horizontals	20	66.7	15	60.0	19	79.2	19	67.9	13	46.4
	Abatidas	8	26.7	10	40.0	5	20.8	9	32.1	11	39.3

**Table 5:** Changes between preoperative and follow-up visits. Global reference lip patterns (Suzuki and Tsushihashi classification), lip thickness and commissures (F) Frequency (%) Percentage.

Change		Preoperative and 15 days		Preoperative and one month		Preoperative and three months		Preoperative and six-months	
	Y/N	(F)	(%)	(F)	(%)	(F)	(%)	(F)	(%)
Suzuky - First quadrant (upper lip - right)	Yes	10	40.0	9	37.5	14	50.0	18	64.3
	No	15	60.0	15	62.5	14	50.0	10	35.7
Suzuky - Second quadrant (upper lip - left)	Yes	9	36.0	9	37.5	15	53.6	15	53.6
	No	16	64.0	15	62.5	13	46.4	13	46.4
Suzuky - Third quadrant (lower lip - left)	Yes	6	24.0	13	54.2	11	39.3	10	35.7
	No	19	76.0	11	45.8	17	60.7	18	64.3
Suzuky - Fourth quadrant (lower lip - right)	Yes	10	40.0	14	58.3	15	53.6	11	39.3
	No	15	60.0	10	41.7	13	46.4	17	60.7
Right upper lip thickness	Yes	11	44.0	12	50.0	13	46.4	11	39.3
	No	14	56.0	12	50.0	15	53.6	17	60.7
Upper left labial thickness	Yes	11	44.0	12	50.0	14	50.0	16	57.1
	No	14	56.0	12	50.0	14	50.0	12	42.9
Lower left labial thickness	Yes	7	28.0	10	41.7	14	50.0	5	17.9
	No	18	72.0	14	58.3	14	50.0	23	82.1
Right lower lip thickness	Yes	8	32.0	11	45.8	10	35.7	12	42.9
	No	17	68.0	13	54.2	18	64.3	16	57.1
Right commissure	Yes	6	24.0	7	29.2	7	25.0	12	42.9
	No	19	76.0	17	70.8	21	75.0	16	57.1
Left commissure	Yes	8	32.0	4	16.7	7	25.0	10	35.7
	No	17	68.0	20	83.3	21	75.0	18	64.3

**Table 6:** Existence of changes between visits grouped by variable type: global reference lip patterns, lip thickness, and commissures.

Variables	Y/N	Frequency	Percentage
Suzuky - Changes between the preoperative visit and the 15-day visit	Yes	16	64.0
	No	9	36.0
Suzuky - Changes between pre-op and one-month visit	Yes	19	79.2
	No	5	20.8
Suzuky - Changes between preoperative visit and three months	Yes	24	85.7
	No	4	14.3
Suzuky - Changes between pre-operative visit and six months	Yes	26	92.9
	No	2	7.1
Lip thickness - Changes between pre-op visit and 15 days	Yes	20	80.0
	No	5	20.0
Lip thickness - Changes between preoperative visit and one month	Yes	19	79.2
	No	5	20.8
Lip thickness - Changes between pre-op visit and three months	Yes	23	82.1
	No	5	17.9
Lip thickness - Changes between pre-op visit and six months	Yes	22	78.6
	No	6	21.4
Commissure - Changes between pre-operative visit and 15 days	Yes	12	48.0
	No	13	52.0
Commissure - Changes between preoperative visit and one month	Yes	11	45.8
	No	13	54.2
Commissure - Changes between pre-operative visit and three months	Yes	13	46.4
	No	15	53.6
Commissure - Changes between preoperative visit and six months	Yes	17	60.7
	No	11	39.3

## Data Availability

The datasets used and/or analyzed during the current study are available from the corresponding author.
